# Instar- and host-associated differentiation of bacterial communities in the Mediterranean fruit fly *Ceratitis capitata*

**DOI:** 10.1371/journal.pone.0194131

**Published:** 2018-03-08

**Authors:** Antonino Malacrinò, Orlando Campolo, Raul F. Medina, Vincenzo Palmeri

**Affiliations:** 1 Department of Physics, Chemistry and Biology (IFM), Linköping University, Linköping, Sweden; 2 Dipartimento di Agraria, Università degli Studi “Mediterranea” di Reggio Calabria, Reggio Calabria, Italy; 3 Department of Entomology, Texas A&M University, College Station, Texas, United States of America; Universita degli Studi di Camerino, ITALY

## Abstract

Microorganisms are acknowledged for their role in shaping insects’ evolution, life history and ecology. Previous studies have shown that microbial communities harbored within insects vary through ontogenetic development and among insects feeding on different host-plant species. In this study, we characterized the bacterial microbiota of the highly polyphagous Mediterranean fruit fly, *Ceratitis capitata* (Diptera: Tephritidae), at different instars and when feeding on different host-plant species. Our results show that the bacterial microbiota hosted within the Mediterranean fruit fly differs among instars and host-plant species. Most of the bacteria harbored by the Mediterranean fruit fly belong to the phylum Proteobacteria, including genera of Alphaproteobacteria such as *Acetobacter* and *Gluconobacter*; Betaprotobacteria such as *Burkholderia* and Gammaproteobacteria such as *Pseudomonas*.

## Introduction

It is widely acknowledged that microorganisms harbored by insects, in particular bacteria, play an important role in their hosts’ biology and natural histories [1–7]. Indeed, microorganisms possess metabolic properties that are often absent in insects, enabling their hosts to: overcome plant defenses [8], tolerate extreme temperatures [9], acquire immunity against their natural enemies [10], survive feeding nutrient-limited diets [11] and detoxify plant defense compounds [12, 13].

Variations in microbiota composition among insects feeding on different host plant species suggest that insect microbiota may play a role in the definition of herbivorous insects’ host ranges [3, 4, 14, 15]. On the other hand, variation in insect diet may influence the microbiota associated with polyphagous insects feeding on different host-plant species [14, 16–18]. In other words, insect-associated microbial communities may be diet-dependent [19], which has been observed, for example, in bumblebees when reared on artificial diet and moved to outdoors [20], among different castes and ages in *Macrotermes gilvus* (Hagen) according to their respective diets [21], and in many other insect species (see Discussion section). In addition, the reorganization of insect microbiota composition across different instars has been investigated for several insect species. In a comprehensive study, changes in gut microbiota were reported in 218 species belonging to 21 insect orders [22].

Tephritid fruit flies are devastating agricultural pests that can impact several different agricultural crops. Among Tephritid fruit flies, the Mediterranean fruit fly (*Ceratitis capitata* Wied.) represents a serious threat to several crops, with worldwide losses amounting to several billion USD [23], and a host range comprising more than 350 plant species [24]. Before this study, the complete structure of the microbiota associated to *C*. *capitata* was poorly known, with surveys mainly dealing with culture-dependent techniques or molecular approaches with low resolution (mainly DGGE) [25–28]. In *C*. *capitata*, variation in gut bacterial communities has been reported between larvae and adults using pyrosequencing [29].

To our knowledge, we are still missing information on the complete microbiota associated to *C*. *capitata*, and in particular we lack information on microbial composition for pupae and at different moments of larval phase. As well, to date, there is no available data on changes in medfly microbiota accordingly to the host plant. Thus, in this study, we characterize the bacterial communities harbored by *C*. *capitata*, across different instars and host plant species, using 16S rRNA gene metabarcoding [30, 31]. We predict that microbial assemblages hosted by *C*. *capitata* will vary across instars, likely supporting the insect’s specific needs at each stage of metamorphosis. Also, we hypothesize an effect of host-plant species on larval microbial community, since insect’s microbiota can adapt to exploit different diets, or the diet itself can have a major effect on insect’s microbial community.

## Materials and methods

### Ethics statement

The study was carried out on private land and the owner of the land gave permission to conduct the study on this site. The study did not involve endangered or protected species.

### Samples for instar-associated microbial community

Sampling was carried out in the rural area of the province of Reggio Calabria, Italy (38.07 N, 15.71 E) during 2015. First instar larvae, 3^rd^ instar larvae, pupae and adults of *C*. *capitata* were collected from orange fruits (*Citrus sinensis*), in order to assess the instar-dependent variation in the bacterial microbiota. First instar larvae were collected directly opening the fruits, 3^rd^ instar larvae were collected waiting for their exit from fruits. A group of 3^rd^ instar larvae were left to pupate and, another group, to become adults in order to collect respective samples. Pupae chosen to become adults were kept inside 30 x 30 x 30 cm plastic cages (Bugdorm-1, Bugdorm, Taiwan) at room temperature (≈25°C), and checked twice per day for adult emergence. Fifteen specimens per instar (1^st^ instar larvae, 3^rd^ instar larvae, pupae and adults) were stored at -80°C until DNA extraction and used to compare *C*. *capitata* microbiota among different instars.

### Samples for host plant associated microbial community

*Ceratitis capitata* specimens were collected during 2015 from figs (*Ficus carica*—July), prickly pears (*Opuntia ficus indica*—September), peaches (*Prunus persica*—July), cherimoya (*Annona cherimola*—November) and orange fruits (*Citrus sinensis*—December), within a 10 km radius from the sampling area indicated above. Fruits of these species were placed on adsorbing paper inside 30 x 30 x 30 cm plastic cages (Bugdorm-1, Bugdorm, Taiwan) at room temperature (≈25°C). Cages containing fruits were checked twice per day for larval emergence. Last (third) instar larvae were collected waiting for their exit from fruits and stored at -80°C until DNA extraction. Seventy-five specimens (15 per host plant) were used to compare *C*. *capitata* microbiota among different fruit species.

### DNA extraction, 16S rRNA gene amplification and sequencing

Specimens were surface sterilized washing them twice in sodium hypochlorite (1%), and twice in ddH_2_O [32]. Total DNA was extracted from whole single specimens using the MoBio PowerSoil Kit (Mo Bio Laboratories, Inc., Carlsbrand, CA, USA) following the manufacturer’s instructions. DNA was subsequently checked for quantity and quality with a Nanodrop 2000 (Thermo Fisher Scientific Inc., Waltham, MA, USA). The bacterial community was characterized targeting the 16S rRNA gene with primers 515f/806rB [31]. PCR reactions were performed in a total volume of 25 μl, containing about 50ng of DNA, 0.5 μM of each primer, 1X of KAPA HiFi HotStart ReadyMix (KAPA Biosystems, Wilmington, MA, USA) and nuclease-free water. Amplifications were performed in a Mastercycler Ep Gradient S (Eppendorf, Hamburg, Germany) set at 95°C for 3 minutes, 98°C for 30s, 55°C for 30s and 72°C for 30s, repeated 35 times, and ended with 10 minutes of extension at 72°C. Reactions were carried out in triplicate, in order to reduce the stochastic variability during amplification [33]. A non-template control in which nuclease-free water replaced target DNA was utilized in all PCR reactions. Furthermore, amplifications were carried out on 3 nuclease-free water samples that were subjected to the same DNA extraction procedure of the other samples, in order to further control for contamination. We didn’t observe any amplification on negative-control samples, and no sequences were retrieved from sequencing. Libraries were checked on agarose gel for successful amplification, and purified with Agencourt AMPure XP kit (Beckman and Coulter, Brea, CA, USA) using manufacturer’s instruction. A second short-run PCR was performed in order to ligate the Illumina i7 and i5 indexes following producer’s protocol, and amplicons were purified again with Agencourt AMPure XP kit. Libraries were then quantified through Qubit spectrophotometer (Thermo Fisher Scientific Inc., Waltham, MA, USA), normalized using nuclease-free water, pooled together and sequenced with the Illumina MiSeq sequencer (Illumina, San Diego, CA, USA), using the MiSeq Reagent Kit v3 600-cycles chemistry following manufacturer’s protocol.

### Data analysis

Demultiplexed forward and reverse reads were merged using PEAR 0.9.1 algorithm using default parameters [34]. Data handling was carried out using QIIME 1.9 [31], quality-filtering reads, binning OTUs using open-reference OTU-picking through UCLUST algorithm, discarding chimeric sequences discovered with USEARCH 6.1, and assigning taxonomy with GreenGenes database using the BLAST method. Singletons, OTUs retrieved in less than 5 samples and those not associated to bacteria were all discarded from the downstream analyses. Unfortunately, at this step we had to discard 4 samples (3 from *A*. *cherimola* and 1 from *O*. *ficus indica*) because they contained a low number of sequences (< 10,000). Furthermore, during the analysis of bacterial taxa, we focused only on OTUs representing >1% of reads, thus eliminating clusters likely originating from contaminant and, consequently, increasing the repeatability of the results [35].

The diversity of microbial communities in our system has been investigated using three diversity indexes: Shannon’s index [36], Chao-1 index [37], and Faith’s phylogenetic diversity (PD) [38]. The last one calculates diversity as measure of branch length of a phylogenetic tree that includes observed taxa. Comparison of diversity indexes among groups (i.e. instar or host plant) was performed using GLM procedure, supported by Tukey’s test for multiple comparisons. Distances between pairs of samples, in terms of community composition, were calculated using a Bray-Curtis dissimilarity index, and then visualized using NMDS procedure. Differences between sample groups were supported by PERMANOVA analysis, also performed in a pair-wise fashion between samples using Bonferroni correction on *P*-values.

Data analysis was performed using R statistical software [39] with the packages vegan [40], phyloseq [41] and picante [42]. Furthermore, DESeq2 [43] was used to highlight OTUs differentially present among different instars of *C*. *capitata*, and when collected from different host fruits.

## Results

### Microbiota variation among *C*. *capitata* instars

**Fig 1:**
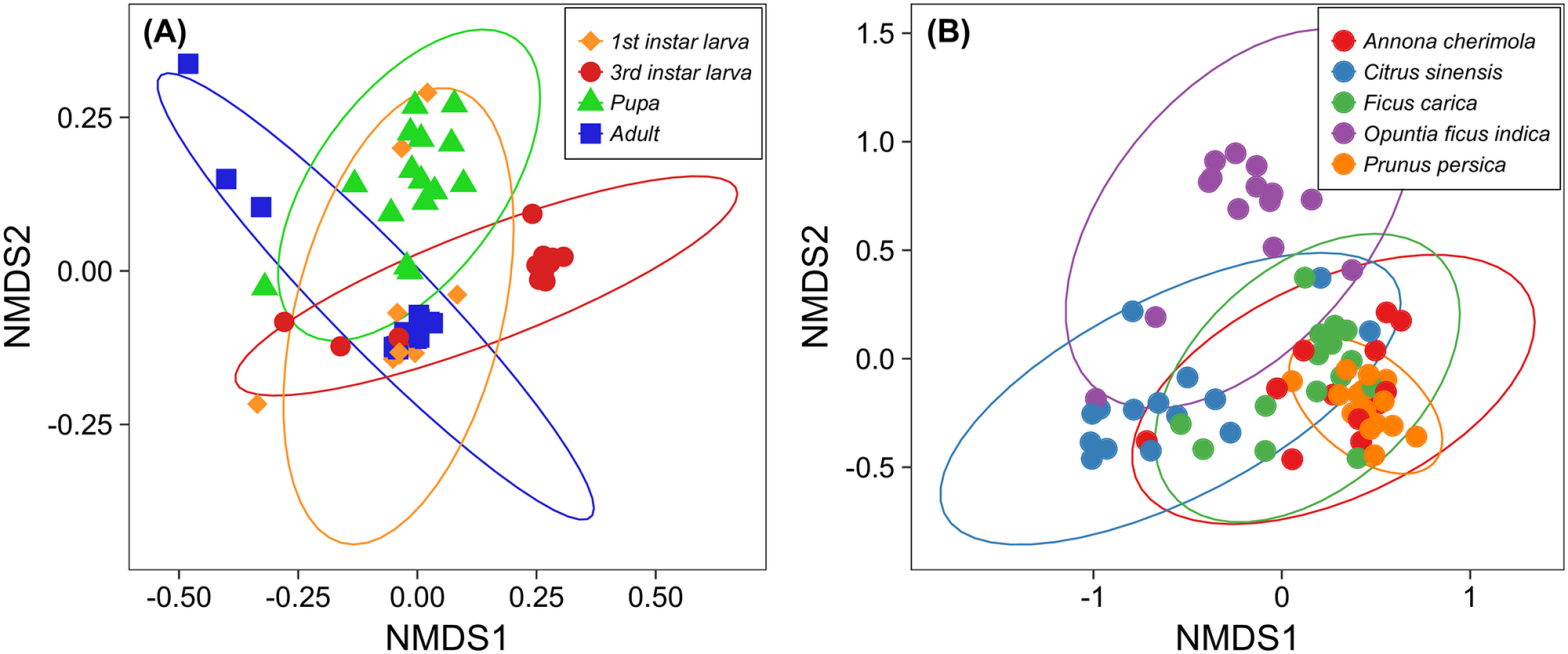
NMDS (Non-metric Multidimensional Scaling) analysis of microbial communities. (A) different life stages of *C*. *capitata* (stress 0.09); (B) larvae of *C*. *capitata* feeding on different host plants (stress 0.16). Ellipses are calculated at the 95% confidence interval.

**Fig 2:**
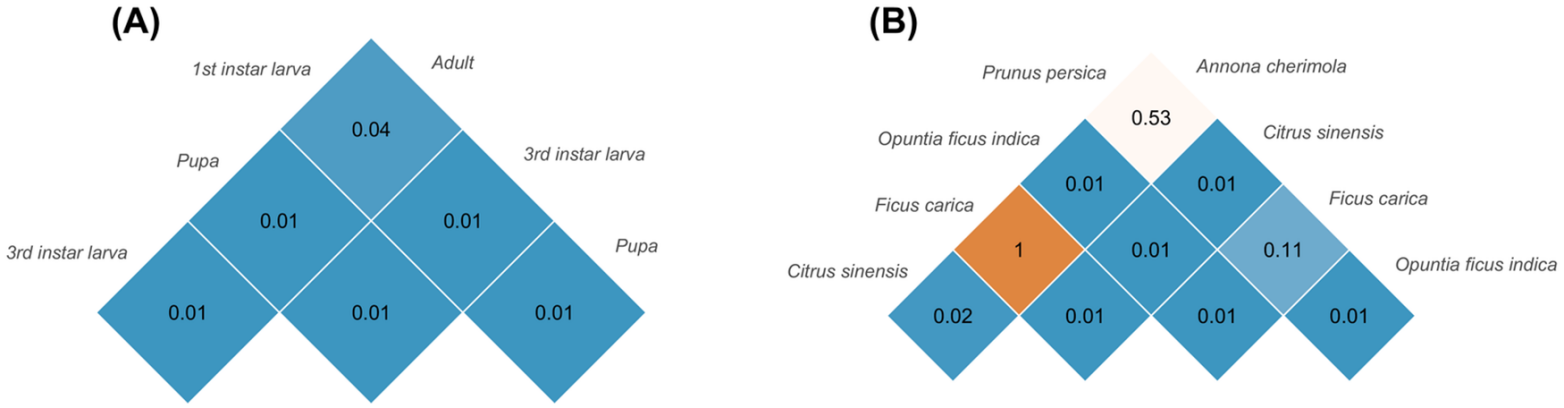
Multiple comparison procedure through PERMANOVA. (A) different life stages of *C*. *capitata*; (B) larvae of *C*. *capitata* feeding on different host plants. Bonferroni-corrected *P* values are reported for each comparison.

Clustering produced 3,169 OTUs that, as suggested by the multivariate PERMANOVA analysis, characterized different microbial communities among the different *C*. *capitata* instars (F_3, 56_ = 6.39, *P*<0.001, stress = 0.09, Fig 1A). A multiple comparison test confirmed that all developmental stages of *C*. *capitata* have a distinct microbial community (Fig 2A). Furthermore, the diversity of the microbial community associated with the 3^rd^ instar larvae and pupal stage was higher compared to the other instars, while the larvae at the first instar had both the lowest Shannon’s diversity and PD indices (Shannon–*P* < 0.001; PD–*P* < 0.05; S1 Table).

Overall, the microbiota associated to *C*. *capitata* was mainly composed by Firmicutes (10.41±2.35%) and Proteobacteria (87.12±2.28%). Within Firmicutes most of the bacterial sequences found in *C*. *capitata* belong to the family Leuconostocaceae (9.59±2.37% of the total bacterial sequences), while Proteobacteria were mostly represented by bacteria belonging to the classes Alphaproteobacteria (16.15±2.09%), Betaproteobacteria (36.36±3.25%), Gammaproteobacteria (29.46±2.51%) and Deltaproteobacteria (5.15±1.15%).

A comparison of bacterial taxa among the different *C*. *capitata* instars (Table 1), as resulted from the differential presence analysis, reveals a pattern of different bacterial associations along *C*. *capitata*’s life history. The microbiota of 1^st^ instar larvae contains a relatively higher abundance of *Burkholderia* compared to the other instars. Later on in the larval phase, the microbial community has a higher representation of *Sphingomonas*, *Pseudomonas* and an unidentified Methylobacteriaceae. Both *Sphingomonas* and the unidentified Methylobacteriaceae were almost absent in the other instars. After pupation, *Leuconostoc* and *Weissella* become relatively more abundant, together with Acetobacteraceae (*Acetobacter* and *Gluconobacter*) and an unidentified Xanthomonadaceae. Except for *Leuconostoc*, the other taxa were retrieved for the other instars at very low abundances. Interestingly, pupae harbored a relatively lower abundance of *Acinetobacter*. Finally, adults, harbored bacteria in the genus *Burkholderia* and an unidentified genus of Deltaproteobacteria in relatively higher abundances.

**Table 1:** Bacterial taxa differentially associated with different instars of *C*. *capitata*.

Taxon	F_4, 66_	*P*	1^st^ instar larvae	3^rd^ instar larvae	Pupae	Adults
*Acetobacter*	15.65	***	n.d.	0.11±0.08% (a)	9.81±2.46% (b)	0.07±0.04% (a)
*Acinetobacter*	7.59	***	17.73±2.74% (b)	14.43±1.59% (b)	5.31±0.61% (a)	14.09±1.85% (b)
*Burkholderia*	10.51	***	57.52±5.16% (c)	18.65±6.57% (a)	25.82±3.37% (ab)	39.56±4.78% (bc)
*Gluconobacter*	24.16	***	n.d.	0.70±0.47% (a)	11.61±2.19% (b)	0.04±0.01% (a)
*Leuconostoc*	7.2	***	9.03±5.77% (ab)	0.99±0.4% (a)	22.69±4.42% (b)	1.3±0.66% (a)
*Pseudomonas*	20.14	***	0.8±0.21% (a)	19.11±3.85% (b)	0.65±0.24% (a)	1.82±0.44% (a)
*Sphingomonas*	22.87	***	0.68±0.06% (a)	9.71±1.8% (b)	0.52±0.17% (a)	0.95±0.14% (a)
Un. Deltaproteobacteria	17.3	***	0.06±0.05% (a)	3.68±1.16% (a)	1.08±0.65% (a)	15.50±3.02% (b)
Un. Methylobacteriaceae	19.61	***	0.72±0.15% (a)	12.12±2.5% (b)	0.3±0.06% (a)	0.86±0.19% (a)
Un. Xanthomonadaceae	5.9	**	0.04±0.02% (a)	0.56±0.14% (a)	1.8±0.6% (b)	0.24±0.1% (a)
*Weissella*	4.32	**	0.52±0.46% (ab)	n.d.	3.71±1.58% (b)	0.04±0.02% (a)

For each instar, it is reported the relative abundance (mean ± SE) of each taxon, with the result of the Tukey’s MCT (different letters on the same row, indicate differences for *P*<0.05). n.d. = not detected

*** *P*<0.001;

** *P*<0.01;

* *P*<0.05;

### Host plant associated microbiota

This analysis yielded a total of 1,118 OTUs. Results from the multivariate analysis on the whole microbial community composition highlighted differences between host plants (F_4, 66_ = 5.83, *P*<0.001, stress = 0.16, Fig 1B). The multiple comparison procedure confirmed what can be visualized on NMDS plot. Indeed, the microbiota of larvae collected from *A*. *cherimola*, *P*. *persica* and *F*. *carica* is very similar, while it is different in comparison to other host plants (*C*. *sinensis* and *O*. *ficus indica* Fig 2B). The diversity analysis (Shannon’s index) highlighted a similar diversity of communities associated to *A*. *cherimola*, *C*. *sinensis*, *F*. *carica* and *O*. *ficus indica* (S2 Table). In the same way, samples from *O*. *f*. *indica* showed the highest phylogenetic diversity, while larvae collected on *P*. *persica* had both the lowest Shannon and PD indices (S2 Table).

The microbiota of *C*. *capitata* 3^rd^ instar larvae was mainly represented by Firmicutes and Proteobacteria. However, the abundance of these phyla varied among flies associated with different host plants. Firmicutes were more abundant on larvae collected from fruits of *A*. *cherimola* (64.4±9.17%), *P*. *persica* (65.33±7.72%) and *F*. *carica* (42.98±7.75%) than *O*. *f*. *indica* (32.06±8.92%) and *C*. *sinensis* (22.09±7.15%) (F_4,66_ = 5.15; *P*<0.01). On the other hand, Proteobacteria were more abundant in *C*. *sinensis* (76.28±7.6%), *F*. *carica* (51.09±8.23%) and *O*. *ficus indica* (57.87±7.83%) than *A*. *cherimola* (31.36±9.54%) and *P*. *persica* (32.46±7.89%) (F_4,66_ = 4.91; *P*<0.01). Bacteroidetes were more abundant in *F*. *carica* (3.11±1.49%) and *O*. *ficus indica* (5.92±1.54%) (F_4,66_ = 4.88; *P*<0.01).

Focusing on differentially abundant taxa (Table 2), it is evident that the microbial community associated to *C*. *capitata* changes when feeding on different plant species. When *C*. *sinensis* was the host plant, most of the microbiota was composed by unidentified Enterobacteriaceae (66.01±9.33%). On *A*. *cherimola* the microbial community was richer in *Lactococcus*, which was rare in *C*. *sinensis* and absent in the other host plants. When feeding on *P*. *persica*, the microbiota of larvae was more abundant in *Leuconostoc* and *Acetobacter*. The latter, was also present in *C*. *sinensis* in low percentage, but was absent in larvae collected from the other hosts. On the other hand, on *F*. *carica* we observed a higher abundance of *Gluconobacter* and *Acinetobacter*. Finally, on *O*. *ficus indica* the changes in microbial associates involved the presence of *Flavobacterium*, *Fructobacillus*, *Azospirillum*, *Pseudomonas*, as well as unidentified Comamonadaceae and Enterobacteriaceae. Other than *Pseudomonas* and unidentified Enterobacteriaceae, all taxa present in the other fruits studies were absent in larvae collected from *O*. *ficus indica*.

**Table 2:** Bacterial taxa differentially associated with larvae of *C*. *capitata* feeding on different host plants.

Taxon	F_4, 66_	*P*	*F*. *carica*	*O*. *ficus indica*	*P*. *persica*	*A*. *cherimola*	*C*. *sinensis*
*Flavobacterium*	7.91	***	0.59±0.28% (a)	3.03±0.86% (b)	0.16±0.15% (a)	0.2±0.11% (a)	0.32±0.17% (a)
*Fructobacillus*	7.53	***	n.d.	27.12±9.6% (b)	n.d.	0.04±0.02% (a)	0.07±0.06% (a)
*Leuconostoc*	13.03	***	39.8±7.34% (bc)	1.71±0.51% (a)	65.03±7.7% (c)	46.25±10.57 (c)	13.75±4.51 (ab)
*Lactococcus*	2.84	*	0.07±0.06% (a)	n.d.	n.d.	14.74±8.84% (b)	2.49±2.4% (ab)
*Acetobacter*	4.36	**	n.d.	0.08±0.05% (a)	16.57±7.06% (b)	0.5±0.42% (a)	1.49±1.23% (a)
*Gluconobacter*	4.06	**	17±6.71% (b)	2.7±2.01% (a)	2.79±1.67% (a)	0.38±0.24% (a)	0.62±0.33% (a)
*Azospirillum*	6.2	***	n.d.	1±0.39%	n.d.	n.d.	n.d.
Un. Comamonadaceae	12.33	***	n.d.	1.1±0.3% (b)	0.03±0.02% (a)	n.d.	0.04±0.03% (a)
Un. Oxalobacteraceae	13.09	***	0.1±0.04% (a)	5.18±1.34% (b)	0.25±0.12% (a)	0.14±0.07% (a)	0.16±0.07% (a)
Un. Enterobacteriaceae	13.2	***	17.76±6.84% (a)	9.64±6.6% (a)	2.23±1.93% (a)	12.1±8% (a)	66.01±9.33% (b)
*Acinetobacter*	2.98	*	4.07±0.83% (b)	3.68±1.02% (ab)	2.3±0.56% (ab)	3.37±0.75% (ab)	0.88±0.36% (a)
*Pseudomonas*	3.53	*	2.61±0.54% (ab)	3.28±0.88% (b)	1.54±0.39% (ab)	3.22±0.85% (b)	0.58±0.21% (a)

For each host plant, it is reported the abundance (mean ± SE) of each taxon, with the result of the Tukey’s MCT (different letters on the same row, indicate differences for *P*<0.05). n.d. = not detected

*** *P*<0.001;

** *P*<0.01;

* *P*<0.05;

## Discussion

In this work, we characterized for the first time the bacterial microbiota harbored by *C*. *capitata* at all insect instars, testing also the hypothesis that diet can have an influence on the structure of larval microbial community. Using a metabarcoding approach we found that each instar of *C*. *capitata* has a different microbial community composition, and that different microbial communities were associated to larvae feeding on different host plants. To our knowledge, few studies have used high-throughput sequencing to explore *C*. *capitata* microbiota [29, 44]. Former studies on medfly mainly used culture-dependent techniques and DGGE (Denaturing Gradient Gel Electrophoresis), however culture-dependent approaches allow the identification of less than 0.1% of microbial diversity [45], while the DGGE provides information about microbial diversity, but its ability to differentiate among taxa is somewhat limited.

We observed a reorganization of gut microbial community at different instars of *C*. *capitata*. Previous studies have reported on the reorganization of gut microbial community of insects across different instars [29, 46–50]. Within the tephritids it has been observed to occur, for example, in *Bactrocera carambolae* Drew & Hancock [51], *Bactrocera dorsalis* (Hendel) [52] and *Bactrocera latifrons* (Hendel) [53]. We found also a higher microbial diversity associated to 3^rd^ instar larvae and pupae, as previously shown to occur in *B*. *dorsalis* [52]. This finds support on the physiology of holometabolous insects, since they undergo through a dramatic anatomical remodeling during development, and often juveniles and adults’ habitats are very different. This microbial reorganization is likely to be the result of the joint interaction by insect host and bacterial symbionts [54]. Interestingly, previous studies [25–28] reported bacteria not retrieved in our analysis (e.g., *Klebsiella* spp., *Pantoea* spp., *Pectobacterium* spp. and *Citrobacter freundii*). This discrepancy could be explained by different factors: (i) former studies on *C*. *capitata* microbiota were conducted in a different geographic area (Israel), thus the formerly reported microbiota may have reflected adaptions to different environmental conditions; (ii) the host plant species used in former studies differ from the ones we used. In our study, bacteria belonging to the genus *Burkholderia* are dominant in young larvae and adults. This bacterial genus has been previously found associated with *C*. *capitata*, but also with *Lutzomyia* sandflies and in several Heteroptera [55–59], and it has been suggested a role in nitrogen fixation when associated with *Tetraponera* ants [60]. At the end of the larval phase, bacteria belonging to the genera *Sphingomonas*, *Pseudomonas* and *Methylobacteraceae* become more abundant in *C*. *capitata*. It is known that *Pseudomonas* can act as an entomopathogen [25], while the function of the other two lineages when associated with insects is currently unknown.

In our study, we also observed the effect of diet on the resulting microbial community of medfly larvae. Previous studies have accounted for the influence of host plant species on the microbiota associated with polyphagous insect species. For example, the microbiota associated with *Acyrthosiphon pisum* Harris [16], *Phylloxera notabilis* Pergande [14], *Helicoverpa armigera* (Hübner) [18], *Thaumetopoea pytiocampa* Den. & Schiff. [61] and *Melitaea cinxia* (L.) [17], has been found to vary when feeding on different host plant species. The shift of the bacterial community according to the host plant can be explained taking into account different aspects. It is, for example, acknowledged that different plant species host a different microbial community [62], so polyphagous insects can just pick a different community when they change host. Furthermore, the diet itself can act on promoting or selecting specific microbial communities, through the interaction with the host. On the other hand, we found that for larvae feeding on *A*. *cherimola*, *F*. *carica* and *P*. *persica*, diet does not have any influence on the microbial community, while it changes when compared to the other host plants (*C*. *sinensis* and *O*. *ficus indica*). In our analysis, *Enterobacteriaceae* seems to play a key role in exploiting *C*. *sinensis* and *O*. *ficus indica* fruits. Previously, Augustinos et al. [63] and Behar et al. [25] reported beneficial effects of *Enterobacteriaceae* on medfly, and Behar et al. [27] suggested a role in nitrogen fixation within medfly guts. *Enterobacteriaceae* were found also to be associated to other tephritids [64, 65, 66]. It has also been postulated that selected pectinolytic and nitrogen-fixing *Enterobacteriaceae* can be vertically transmitted from one generation to the next in frugivorous insects [28]. Interestingly, when feeding on *O*. *ficus indica* and *C*. *sinensis*, larval microbiota is enriched of microbial species which may provide nitrogen-fixation and improve sugar metabolism and pectinolitic activity. Similarly, bacteria belonging to the genus *Acinetobacter* were found in larvae collected from fruits of *F*. *carica*, together with OTUs identified as *Gluconobacter*, which could help insects in detoxifying phenolic glycosides [67] or during nutritionally low periods [68]. Furthermore, *Acetobacter*, which is more abundant in larvae thriving on *P*. *persica*, could have the same function, together with *Leuconostoc* which role is still undisclosed. Also, *Lactococcus* could contribute insect nutrition on *A*. *cherimola*. These results may support the hypothesis that in *C*. *capitata*, and perhaps in other polyphagous species, the insect’s microbial community is the result of an adaptation of insects’ microbiota to a specific diet. On the other hand, it has been reported that the microbial community of some polyphagous species is not affected by diet [69]. Therefore, more work needs to be done to clarify this aspect especially extending the analysis to the host plant microbiota, in order to confirm whether insect’s microbial community adapts to a specific host plant, or if it is simply a subset of plant microbiota.

In a wider view, our results represent an important step in understanding the microbiota associated with the medfly, and its interaction with the host. More generally, it is important for agricultural and forest pests, since shifts in microbial community can extend their dietary range [8, 11, 70], and influence their pest status [4]. The knowledge of microbial associates of pests is thought to be critical for future pest management approaches, which could consider the targeted manipulation of the microorganisms associated to target insects [4, 71–73]. A targeted manipulation of bacterial communities harbored within insect pests could be used to enhance IPM programs, to counteract the spread of insect-borne pathogens and their vectors and to protect beneficial insects [74, 75]. For example, it has been shown that *Wolbachia* can induce cytoplasmic incompatibility in medfly, and it has been resulted successful in pest control under laboratory conditions [76]. Furthermore, in two different moth species, it has been shown that the gut microbial community is essential to *Bacillus thuringiensis* to perform insecticidal activity [77, 78].

In conclusion, our observations show that the microbial community associated to *C*. *capitata* is subjected to substantial changes across all instars, that might suggest a role in fulfilling specific needs during insects’ development. Furthermore, we observed that different host plant species promote variation in microbiota’s diversity associated with medfly larvae. A clear understanding of the mechanisms underlining changes in microbiota composition of polyphagous species according to their diet, are important aspects to understand the biology of insect pests but, as well, could represent the basis of future pest management programs.

## Supporting information

S1 Table Diversity analysis of microbial community associated with different instars of *C*. *capitata*. (DOCX)

S2 Table Diversity analysis of microbial community associated larvae of *C*. *capitata* feeding on different host plants. (DOCX)
