# Supplementary material for: Instar- and host-associated differentiation of bacterial communities in the Mediterranean fruit fly Ceratitis capitata
Source: PLoS One. 2018 Mar 8;13(3):e0194131. doi: 10.1371/journal.pone.0194131 (PMC5843337; doi:10.1371/journal.pone.0194131)
Supplement: S1 Table — (DOCX) [file pone.0194131.s001.docx]

**S1 Table.** **Diversity analysis of microbial community associated with different instars of *C. capitata*.**

| **Stage** | **Shannon** | **Chao1** | **Phylogenetic diversity** |
| --- | --- | --- | --- |
| *1st instar larva* | 1.84±0.07 (c) | 580.6±23.4 (a) | 9.46±0.26 (b) |
| *3rd instar larva* | 2.63±0.14 (a) | 526.9±41.2 (a) | 9.72±0.71 (ab) |
| *Pupa* | 2.62±0.06 (a) | 711.7±21.1 (b) | 11.61±0.41 (a) |
| *Adult* | 2.21±0.09 (b) | 557.3±32.7 (b) | 9.88±0.56 (ab) |
| *F* (df 3, 56) | 14.69 | 7.04 | 3.6 |
| *P* | *** | *** | * |

For each instar and host plant, diversity indices are reported as mean ± SE, together with the result of the Tukey’s MCT (different letters on the same row, indicate differences for *P*<0.05).

*** *P*<0.001; ** *P*<0.01; * *P*<0.05
