# Supplementary material for: Instar- and host-associated differentiation of bacterial communities in the Mediterranean fruit fly Ceratitis capitata
Source: PLoS One. 2018 Mar 8;13(3):e0194131. doi: 10.1371/journal.pone.0194131 (PMC5843337; doi:10.1371/journal.pone.0194131)
Supplement: S2 Table — (DOCX) [file pone.0194131.s002.docx]

**S2 Table.** **Diversity analysis of microbial community associated larvae of *C. capitata* feeding on different host plants.**

| **Host** | **Shannon** | **Chao1** | **Phylogenetic diversity** |
| --- | --- | --- | --- |
| *Annona cherimola* | 1.59±0.17 (ab) | 98.6±9.73 (b) | 3.16±0.17 (bc) |
| *Citrus sinensis* | 1.76±0.09 (ab) | 163.27±12.87 (a) | 3.79±0.19 (b) |
| *Ficus carica* | 1.84±0.12 (ab) | 115.52±7.69 (b) | 3.34±0.22 (bc) |
| *Opuntia ficus indica* | 2.15±0.29 (a) | 169.93±13.33 (a) | 5.32±0.34 (a) |
| *Prunus persica* | 1.37±0.11 (b) | 77.61±8.85 (b) | 2.52±0.16 (c) |
| *F* (df 4, 66) | 2.967 | 14.24 | 21.66 |
| *P* | * | *** | *** |

For each instar and host plant, diversity indices are reported as mean ± SE, together with the result of the Tukey’s MCT (different letters on the same row, indicate differences for *P*<0.05).

*** *P*<0.001; ** *P*<0.01; * *P*<0.05
